# Assessment of validity of a high-yield surface electromyogram decomposition

**DOI:** 10.1186/1743-0003-10-99

**Published:** 2013-09-23

**Authors:** Xiaogang Hu, William Z Rymer, Nina L Suresh

**Affiliations:** 1 Sensory Motor Performance Program, Rehabilitation Institute of Chicago, 345 E Superior Street, Room 1378, Chicago, IL 60611, USA; 2 Department of Physical Medicine and Rehabilitation, Feinberg School of Medicine, Northwestern University, Chicago, IL, USA

**Keywords:** Surface electromyogram, Motor unit, sEMG decomposition, Validation, Spike triggered averaging

## Abstract

**Background:**

Using recordings from a five-pin surface sensor array, a template-based surface electromyogram (sEMG) decomposition system has been developed to identify single motor unit discharge properties. However, the reliability of such template based decomposition results has not been thoroughly examined except by the developers. The focus of this study was to assess the validity of the motor unit decomposition technique, using EMG recordings from the first dorsal interosseous muscle of able-bodied human subjects.

**Methods:**

Two tests were utilized. In the first test, a spike triggered averaging (STA) analysis was used to derive motor unit action potential (MUAP) parameters. We examined these STA derived MUAP shapes after firing times were perturbed by added timing noise. In the second test, a cross-correlation analysis was performed between the sEMG signal and MUAP trains constructed using STA estimates and their firing times.

**Results:**

In the first test, we found that MUAP shape features deteriorated significantly when rather small (0.6-2 ms) timing errors were added, affirming that the decomposed firing times are presumptively valid. The results of the second test reveal that the cross-correlation index between the EMG and MUAP trains increased monotonically up to 0.71 when the identified MUs were progressively added to reconstructed MUAP trains; however, this increment disappeared when the firing times or the MUAP templates were shifted randomly.

**Conclusions:**

Based on our STA selection criteria, our results suggest that the firing times and estimated MUAP shapes for each MU generated by the decomposition algorithms are presumptively valid.

## Background

Single motor unit (MU) firing properties provide valuable information about the neuromuscular system and are widely used in physiology [1], motor control [2], and clinical [3] fields. Intramuscular electromyogram (EMG) recordings are typically used to derive single MU activities. However, this intramuscular technique has several limitations. Intramuscular recordings can detect only a limited number of MUs at any given force level, data collection and analysis is time consuming, and electrode insertion can be painful. In contrast, non-invasive recordings capable of extracting single motor unit activities from the overlying skin surface have been developed. With further development of high-speed computing techniques, various methods of MU decomposition have been proposed, where single MU firings can be extracted from intramuscular EMG or surface EMG (sEMG) interference patterns using a range of decomposition algorithms [4-9]. In our laboratory, we have focused on testing a novel sEMG recording and decomposition system [10]. The associated decomposition algorithm represents an advance over earlier intramuscular MU decomposition methods [6,11], and there have also been several innovative updates in sEMG decomposition methodology since its inception [12,13]. The output of this decomposition algorithm provides the firing times and action potential templates of a large number of MUs extracted from the sEMG during a muscle contraction over a large force range. Although the approach is promising, the accuracy of the decomposition results has not been determined by sources outside of the developers, and the overall validity of the approach has also been questioned [14]. Accordingly, the objective of the current study was to assess the overall validity of this sEMG decomposition algorithm.

In earlier studies, the accuracy of the decomposition results has been assessed using a reconstruct-and-test approach by De Luca and colleagues [10,15]. Specifically, the EMG signal was decomposed into constituent MU action potential (MUAP) trains. A simulated EMG signal was then constructed based on the decomposed unit firing times and action potential templates, and the simulated signal was decomposed again. The decomposition results of the recorded and simulated EMG signals were then compared to assess the accuracy of the algorithm. In another validation protocol, the MU firing times were randomized during the reconstruction of EMG signals to further assess the decomposition results [16]. Although these validation methods were based on simulated data that inevitably contain simplifications compared with real EMG data, the MUAPs and firing statistics were derived from realistic EMG signals and thus represent realistic characteristics of observed EMG signals [10,15]. However, neither of these validation methods have been universally accepted (see the discussions between [14] and [17]).

To address these concerns, the objective of this study was to employ two sets of tests to examine and assess the validity of decomposition results of the sEMG signal recorded from the first dorsal interosseous (FDI) muscle. We devised indirect methods of assessment, because the recorded signal consisted of a large number of motor units with a significant amount of superposition.

In the first set of tests, a spike triggered averaging (STA) technique was used to reconstruct the action potential shape [18-20]. This technique uses the decomposition-based MU firing times as triggers for the recorded raw sEMG signal. The formation of the decomposition system template shape starts from uncontaminated instances of the basic MUAP shape in the recorded EMG record. The template is then matched across all viable MUAP candidates in the EMG signal [6,11]. The template is a weighted average of the variations of the MUAP shape across the EMG record. In contrast, the STA derived template is working backwards using the MU event times to derive the same MUAP shape as provided by the decomposition system. The assumption is that if the system identified MU shapes do occur at the system identified MU event times, then we will be able to extract the same shape using STA.

The STA MUAP estimate was used for further analysis to derive decomposition related parameters. Two STA features were extracted: (1) the correlation between the STA estimates and the decomposition templates, and (2) the waveform stability of the STA estimates. Gaussian noise was then added to perturb the timing of the originally decomposed firings in the STA analysis. We hypothesized that features derived from the STA analysis would show substantial changes (i.e., the STA would not fit with the decomposition estimates and the waveform would show high variation) if the decomposed firing times are largely correct. Conversely, if the firing times are already erroneous from the decomposition, we would expect little changes in the STA features.

The second set of assessments involves a cross-correlation analysis between the raw EMG signal and the MUAP trains constructed using the STA estimates. Reliably decomposed MUs were selected and MUAP trains were constructed using the firing times and the STA estimates. The MUAP trains from different MUs were combined linearly, after which the combined MUAP trains were correlated with the recorded raw EMG signal. We hypothesized that the cross-correlation would increase with the number of MUAP trains added together, if the firing times were correct and the STA estimates represent the true MUAPs. Additionally, if the firing times or the MUAP templates were perturbed (i.e., the raw EMG did not contain the reconstructed random MUAP trains), we would expect low cross-correlations.

Using these tests we were able to validate the decomposition results of the sEMG recorded from the FDI muscle. The outcome of our first assessment showed that the MUAP shape features of the STA estimates were lost even if a small (0.5-2 ms) amount of timing noise was added to the firing times. The second assessment, the cross-correlation index between the raw EMG and MUAP trains increased monotonically with an increasing number of MUs added to the simulated MUAP train. This incremental change was absent when the firing times or the MUAP templates were shifted randomly. The results of our analysis provide support for the validity of surface EMG decomposition systems, and also provide a potential tool for routine assessment of the output of general MU decomposition algorithms for surface EMG.

## Methods

### Participants

Eight right hand-dominant neurologically intact individuals (4 male, 4 female) volunteered to participate in this study. The EMG activity of the FDI muscle was recorded during isometric abduction of the right index finger. The participants gave informed consent via protocols approved by the Institutional Review Board under the Office for the Protection of Human Subjects at Northwestern University.

### Experimental setup

Participants were seated upright in a Biodex chair with their upper arm resting on a support. To standardize hand position and to minimize contributions of unrecorded muscles, the forearm was immobilized in a brace and placed in a ring mount interface attached to an elbow rest. The forearm was placed in full pronation and the wrist was held neutral with respect to flexion/extension. The little, ring and middle fingers were extended away from the index finger and strapped to the support surface. The thumb was secured at an approximately 60 degree angle to the index finger. The index finger was placed in line with the 2^nd^ metacarpal and the long axis of the forearm creating a neutral MCP joint angle. The proximal phalanx of the index finger was cast and fixed to a ring-mount interface attached to a six degrees-of-freedom load cell (ATI, Inc.). The recorded forces from the abduction/adduction direction were low pass filtered (cutoff =200 Hz) and digitized at a sampling frequency of 1 kHz. The subjects were instructed to produce required abduction forces while minimizing the off-axis forces.

#### EMG recordings

**Figure 1:**
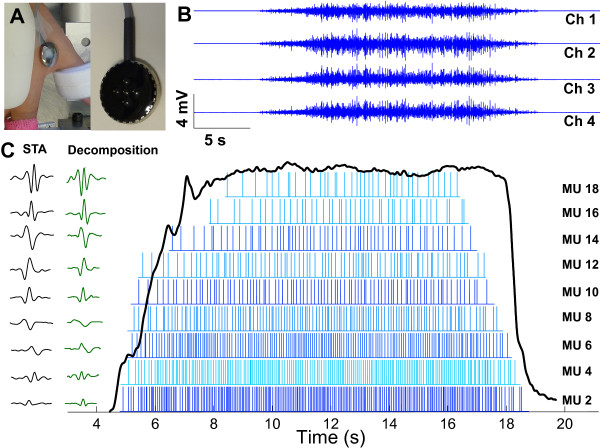
**Surface EMG recording and decomposition. A**: 5-pin sEMG sensor array. **B**: 4 channels of sEMG recorded from the first dorsal interosseous muscle. **C**: Decomposed firing spikes and action potential templates of respective MUs. The STA estimated action potentials are also shown for comparison. The force trajectory is overlaid on top of the firing spikes.

The subject’s skin was prepared by cleaning the superficial layers with adhesive tape, and the skin was then cleaned with alcohol pads to ensure proper electric contact and low baseline noise. sEMG was recorded from the FDI (Figure 1A) using a surface sensor array (Delsys, Inc.) that consisted of 5 cylindrical probes (0.5 mm diameter) as shown in Figure 1A. The probes are located at the corners and at the center of a 5 × 5 mm square. Pairwise differentiation of the 5 electrodes yields 4 channels of sEMG signals (Figure 1B). The sEMG sensor and a reference electrode were connected to 4 channels of a Bagnoli sEMG system (Delsys, Inc.). The signals were amplified and filtered with a bandwidth of 20 Hz to 2 kHz. The signals were sampled at 20 kHz and stored on a computer for decomposition processing.

### Procedures

For the first trial of the experimental session, subjects were asked to perform maximal voluntary contractions (MVCs) for 3 s. This maximum contraction was repeated 3 times in total, with 1 min rest between trials. The largest value of the 3 trials was designated as the MVC. The rest of the session consisted of a series of isometric voluntary contractions during which the subject was asked to follow trapezoid force trajectories displayed on a computer screen, each at a different varying percentage of the MVC. The force output in one exemplar trial is shown in Figure 1C (the black trace). The trapezoid trajectory contains 5 segments: a 5 s quiescent period for baseline noise calculation, an up-ramp increased at a rate of 10% MVC/s, a constant force of the prescribed % MVC for 12 s, a down-ramp decreased at 10% MVC/s, and a 3 s quiescent period. For the main part of the experiment, the subjects performed 4 blocks of trials with five repetitions of each block. Four constant force levels (20%, 30%, 40%, and 50% MVC) were tested, and each block contained one force level. The order of the force levels was randomized for each subject. A 30 s rest period between repetitions was provided.

### Decomposition algorithm

In order to clarify the relation between the decomposed MUAP templates and the STA derived MUAP shapes, a brief summary of template formation and matching used in the decomposition algorithm follows.

The decomposition algorithm consists of two stages. The first stage involves MUAP template creation, matching, and updating [10]. In the first step the algorithm identifies as many template shapes as possible from all possible candidates derived from the original continuous signal. The algorithm searches for occurrences of uncontaminated shapes, which are then used to initiate a MU template. Second, matching of the MUAP templates goes through a maximum a posteriori probability classifier that uses the information of the correlation between the templates and the EMG signal, the amplitude of action potential, and the remaining energy of the EMG signal. Lastly, variations in the shape of the MUAP template (as identified through the matching procedure) are documented as different versions of the same template. The output to the user is a weighted average of the different variations of MUAP templates. The decomposed MUAP templates (Figure 1C) provide information regarding the MUAP shape, but not necessarily the actual amplitude voltage of the MUAP because of the weighting process.

The second stage identifies MUAPs within complex superpositions in the EMG signal. Multiple MUAP templates are iterated through a discrimination analysis to calculate the combination of MUAP templates that best matches the signal shape while also satisfying the criteria that the coefficient of variation of the inter-spike intervals (ISIs) of these MUs is at a minimum. It is worth noting that not all the MUAPs embedded in the EMG signal are identifiable, especially for those small MUAPs that are blurred by background noise and extensive superposition. The decomposition system output consists of the MUAPs that were most reliably identified, and the algorithm is not residual based. Thus the residual signal varies according to the recording circumstances, including experimental protocol. Detailed information for the decomposition algorithm is described in [10].

### Spike triggered averaging derived MUAP features

Spike triggered averaging (STA) analysis aligns segments of EMG signals based on MU firing events. This method provides the ability to attenuate non-time-locked activities from the complex interference pattern and extract action potentials related exclusively to the triggering events. In this study, STA analyses were performed to assess the stability of the MUAP that include:

1) The correlation between the STA and the decomposed action potential templates provided by the decomposition algorithm. A comparison of the STA and the decomposed templates is shown in Figure 1C.

2) The variability in the STA derived MUAP peak-peak amplitude across epochs/segments of the EMG signal. The stability of the MUAP peak-peak amplitude, instead of the entire MUAP waveform, was assessed, because the MUAP amplitude provides key information about the physiological properties of the MU (e.g., MU size).

The STA analysis of sEMG involved three stages of processing.

First, the STA was performed on the individual four sEMG channels. The firing times identified from the decomposition system were used as triggers for the STA calculation. This resulted in one representative waveform estimate for each algorithm identified MU in each channel (Figure 1C). The time interval used to derive the action potential estimate was set at 10 ms prior to and after the estimated firing time; the firing time was placed at the algebraic center of the time window. The MU firing events of the entire trial were used for the MUAP estimation.

Second, two separate STA features were calculated to determine which MUs would be retained for EMG reconstruction. These features were designed to assess the stability of the waveform over the trial duration.

For the first feature, the STA estimated MUAP was calculated based on a window (EMG segment) length of 4 s, and the MUAP variation across segments was then calculated. This window yielded approximately 50–100 firing events in each window, and the window was shifted over the sEMG signal, using a step size of 0.5 s. The number of steps ranged from 24 to 36 depending on the force level and recruitment threshold of individual MUs. The coefficient of variation (CV: standard deviation normalized by the mean) of the peak-peak (P-P) amplitude of the MUAP templates, for each step change, was calculated as a measure of the stability of the waveform average. The P-P amplitude was the difference between the maximal positive and negative peaks of the MUAP.

For the second feature, the MUAP estimates derived using the STA method were compared with the decomposition based templates (a 20 ms segment). The maximum linear correlation coefficient between the STA estimate (calculated over the entire trial duration) and the decomposition estimated templates was computed as a second measure of the reliability of the waveform average. The STA derived template was shifted 10 ms backward and forward one data sample at a time (0.05 ms sample interval) relative to the decomposition template to find the maximum correlation coefficient. The decomposition estimated templates are derived via a weighted average of the various templates used to represent each MU; the STA estimates were calculated directly from the raw EMG data. A high correlation between these two estimates signifies the reliability of the STA waveform average.

Third, the calculated features of the STA estimates were used as criteria to select MUs. Specifically, the MUs with CV of P-P amplitude < 0.2 and a correlation coefficient > 0.7 were selected for later calculation. The average CV and average correlation coefficient across the 4 channels was calculated provided that the 4 channels give rise to similar values of these features.

### STA calculations using altered MU firing times: validation of identified firings

If the action potentials are correctly identified, a disturbance of the timing of firings should significantly change the STA waveform, because it is calculated from the identified firing times for each MU. Conversely, if the firing times are already incorrectly identified, disturbed firing times should have little effect on the STA features (i.e., P-P amplitude and CV values).

**Figure 2:**
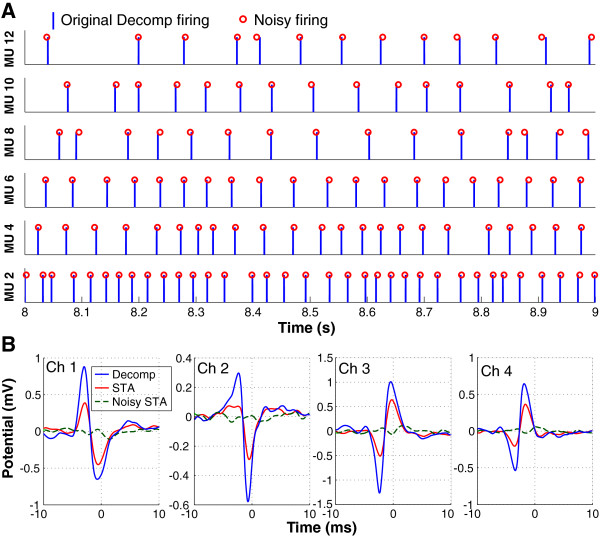
**Noisy firings and comparison of action potential shapes. A**: Firing spikes of exemplar MUs. The vertical bars represent the originally decomposed firing events, and the circles represent the firing times with added Gaussian noise (*SD* = 0.1 * *SD* of ISIs). **B**: A comparison of action potential estimates from 4 channels of EMG signals.

To test this hypothesis, different levels of Gaussian noise (each generated independently) were added to the identified firing times of each MU such that each individual firing time is shifted randomly. Specifically, the noise had a zero mean and a standard deviation (SD) that was scaled with the SD of the ISIs of MU firings. The scaling of the SD ranged from 0 to 0.6 with an increment of 0.1. The exemplar MU spike trains (original decomposition spike train denoted by vertical bars vs. noisy spike train denoted by circles) are shown in Figure 2A. In this example, the scaling factor of the SD of the noise was set at 0.1. The SD of ISI of these MUs ranged from 6 to 18 ms. Consequently, the SD of the added firing noise ranged from 0.6 to 1.8 ms. The STA analysis was then performed based on the firing times with noise added. A comparison of the decomposed action potentials from 4 channels of an exemplar MU, the STA estimates using the original firing times, and the estimates using noisy firing times are shown in Figure 2B. The STA features (correlation between the STA and decomposition templates as well as the CV of MUAP amplitude) were calculated to examine the influence of possible timing errors on STA estimates. During this set of assessment, all the decomposed MUs were used.

### Cross-correlation validation of the identified firings and template shapes

When the interference sEMG patterns are decomposed into constituent MUAP trains, each MUAP train contains key information about the EMG signal, provided that the firing times are identified correctly, and the estimated MUAP templates represent the true MUAP shape. Therefore, one would expect measurable correlation between the interference EMG signal and MUAP train derived from the decomposed MUs and firing times, and this correlation should become stronger when more MUAP trains are combined together. Conversely, if the identified firing times or the estimated MUAPs did not match the true firings and the true MUAP shapes of the MUs that constitute the EMG signal, we would expect a low correlation between the EMG signal and the combined MUAP trains of multiple MUs.

We performed a cross-correlation analysis to test this hypothesis. This analysis consists of several steps:

1) MUs with stable STA derived MUAP shapes were selected based on the STA features. Specifically, the MUAPs with CV of P-P amplitude < 0.2 and a correlation coefficient > 0.7 were selected for later calculation.

2) MUAP trains were constructed using the firing times of the selected MUs and the STA derived MUAPs.

3) MUAP trains of different MUs were combined *one at a time* and the cross-correlation between the combined MUAP trains and the raw EMG was calculated. The MUAP trains were combined in two separate ways (from the first to the last recruited MUs and from the last to the first recruited MUs) to examine the order effect on the cross-correlation.

4) To further validate the identified firing times, Gaussian noise (a zero mean and *SD* = 0.1 * *SD* of ISIs) was added to the identified firing times to disturb the timing. We expect a low cross-correlation, if the original firing times were correctly identified.

5) To further validate the estimated MUAP shapes, instead of using the original STA MUAP template, we randomly assigned a template from other MUs and constructed the MUAP trains. We expect a reduced cross-correlation between the random MUAP train and the EMG signal, if the original STA templates were estimated accurately.

In summary, we examined 4 ways of combining the MUAP trains when performing the cross-correlation with the raw EMG signal:

1) Using original firing times and original STA templates and combining MUAP trains from the first to the last recruited MUs (MU index from small to large (S-L)).

2) Using original firing times and original STA templates and combining MUAP trains from the last to the first recruited MUs (MU index from large to small (L-S)).

3) Construct MUAP trains using *noisy* firing times and original STA templates.

4) Construct MUAP trains using original firing times and *randomly shuffled* STA templates.

## Results

### Influence of ‘noisy’ firing times on STA correlation

**Figure 3:**
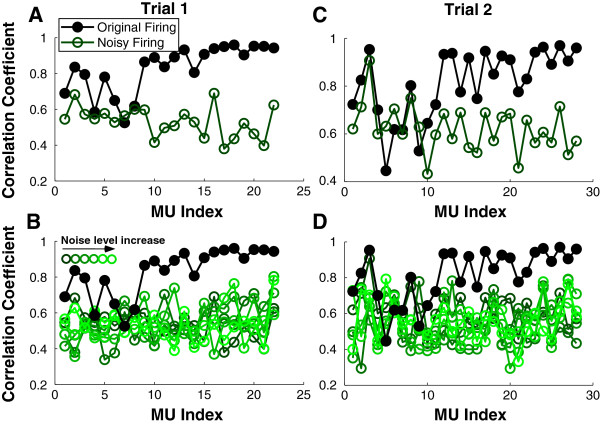
**Correlation coefficients between the STA and the decomposition estimates. A**: The correlations between the EMG and STA estimates using the original firings are shown in filled circles. The correlations calculated based on noisy firings (*SD* of noise = 0.1 * *SD* of ISIs) are shown in open circles. **B**: The correlations calculated based on noisy firings (*SD* of noise = *n* * *SD* of ISIs) are shown in open circles. The noise level increment (*n* increased from 0.1 to 0.6) was color coded. The correlation results of a second trial are shown in **C** and **D** using the same symbol notation as in trial 1.

We examined the impact of imposed Gaussian timing shifts of firing times on the correlation between the STA and the decomposition-derived templates. Figure 3 illustrates the changes in the correlation between the STA and the decomposition estimates when the timing of firing was disturbed. Figure 3A shows the correlations of an exemplar trial at 40% MVC steady hold. The MU index was ordered based on recruitment, and each symbol represents an individual MU. As shown in the graph, most of the MUs had correlations (shown in filled symbols) higher than 0.7 when the STA was estimated using the originally decomposed firing times. In contrast, the correlations (shown in open symbols) were reduced substantially when Gaussian noise (*SD* = 0.1 * *SD* of ISIs) was added to the firing times. When different amounts of firing noise was added (Figure 3B), the correlations (open symbols) remained low regardless of the noise level.

To our surprise, the correlations did not show a systematic change with the amount of noise added. Additionally, the noise had little effect on some MUs; namely, the correlations remained more or less the same at different noise levels in those MUs (MU 4, 6, 7, and 8) that already had low correlations between decomposition and STA estimates using the original firings.

The STA correlation of a second exemplar trial at 50% MVC is shown in Figure 3C. Similar to the first trial, most analyzed MUs had correlations (filled symbols) higher than 0.7, and the correlations (open symbols) were also reduced substantially when a small amount of noise (*SD* = 0.1 * *SD* of ISIs) was added. The different noise levels (Figure 3D) had similar influence on the correlation results. Because the firing times are shifted in a stochastic way (Gaussian distribution with zero mean), the actual timing shifts may be minimal for some MU (e.g. MU 3 in Figure 3C); as a result, the change of correlation is minimum.

A paired *t*-test was performed on the difference in the correlation coefficients between the original and noisy firing condition for individual trials. The average correlation was calculated across different noise level conditions, given the similar noise influence. Because the correlation values are bounded by 0 and 1, a logit transformation was performed on the correlation prior to the *t*-test. As shown in Table 1, the reduction of correlation due to timing shift was significant (*p* < 0.05) for all 8 trials of all the 8 subjects.

**Table 1:** P-values of t-tests on the correlation coefficient results

**%MVC**	**20%**	**20%**	**30%**	**30%**	**40%**	**40%**	**50%**	**50%**
**Subject 1**	7.51E-06	4.32E-06	4.07E-07	1.51E-04	1.06E-05	3.12E-06	1.01E-06	2.66E-06
**Subject 2**	1.31E-08	9.74E-08	1.42E-09	8.57E-08	3.59E-06	2.32E-07	1.23E-08	2.38E-08
**Subject 3**	3.60E-10	6.02E-10	9.22E-11	6.27E-09	5.68E-10	1.48E-05	2.28E-05	2.29E-07
**Subject 4**	5.45E-12	1.58E-10	2.63E-12	2.73E-09	4.78E-07	2.30E-10	9.30E-09	5.36E-10
**Subject 5**	1.54E-07	1.01E-07	2.12E-08	1.17E-05	5.49E-07	1.49E-07	6.78E-08	6.95E-08
**Subject 6**	2.67E-07	4.66E-08	7.45E-07	8.32E-08	4.15E-07	4.05E-06	1.24E-07	2.90E-08
**Subject 7**	1.28E-07	2.27E-07	4.69E-08	2.29E-08	1.36E-08	3.97E-09	2.36E-06	3.22E-06
**Subject 8**	4.78E-08	3.08E-12	4.76E-10	3.63E-11	1.72E-09	1.60E-07	5.44E-08	9.67E-09

### Influence of noisy firings on STA shape variation

**Figure 4:**
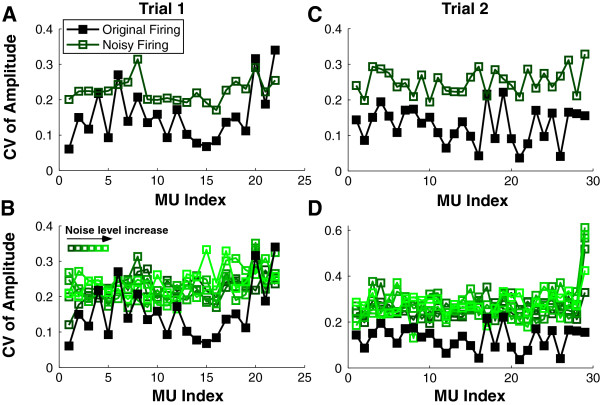
**CV of P-P Amplitude of the STA estimates. A**: The CVs of amplitude using the original firings are shown in filled squares. The CVs calculated based on noisy firings (*SD* of noise = 0.1 * *SD* of ISIs) are shown in open squares. **B**: The CVs calculated based on noisy firings (*SD* of noise = *n* * *SD* of ISIs) are shown in open squares. The noise level increment (*n* increased from 0.1 to 0.6) was color coded. The CV results of a second trial are shown in **C** and **D** using the same symbol notation as in trial 1.

Figure 4 displays the changes in the CV of P-P amplitude of the STA MUAP estimates (same trials as in Figure 3) when the timing of firing was disturbed. Figure 4A shows the CV results of an exemplar trial at 40% MVC. As shown in the graph, most of the MUs had CVs (filled symbols) lower than 0.2, when the STA derived MUAP template was estimated using the original firing times. In contrast, the CVs (open symbols) increased substantially (larger than 0.2) when a small amount of noise (*SD* = 0.1 * *SD* of ISIs) was added to the firing times. Similar to the correlation results, when different amount of firing noise was added (Figure 4B), the CVs (open symbols) remained high (more or less at the same level) regardless of the noise level.

The CV of the MUAP P-P amplitude of a second exemplar trial at 50% MVC is shown in Figure 4C. As for the first trial, most MUs had CVs (filled symbols) lower than 0.2, and the CVs (open symbols) also increased substantially when a small amount of noise was added. The different noise levels (Figure 4D) had similar influence on the CV values. A paired *t*-test was performed to examine the changes of CV (original vs noisy firing condition). The average CV was calculated across different noise level conditions. Because CV values are lower bounded by 0, a logarithmic transformation was performed prior to the *t*-test. As shown in Table 2, the increment of CV due to noisy firings was significant (*p* < 0.05) for the 8 trials of all the 8 subjects.

**Table 2:** P-values of t-tests on the correlation coefficient results

**%MVC**	**20%**	**20%**	**30%**	**30%**	**40%**	**40%**	**50%**	**50%**
**Subject 1**	1.23E-03	1.83E-04	2.88E-03	7.18E-05	1.15E-04	6.83E-06	6.27E-03	2.70E-07
**Subject 2**	5.71E-08	1.55E-04	1.92E-10	3.29E-06	6.40E-07	5.87E-07	3.68E-09	2.31E-07
**Subject 3**	1.54E-04	3.61E-07	4.57E-08	1.19E-05	7.27E-04	6.17E-03	1.46E-06	1.38E-03
**Subject 4**	9.48E-10	5.03E-04	1.85E-06	1.53E-06	2.48E-05	4.26E-06	2.84E-04	1.07E-02
**Subject 5**	1.84E-06	1.80E-04	6.26E-05	8.08E-06	5.64E-06	3.11E-06	5.49E-08	6.02E-07
**Subject 6**	4.95E-05	9.80E-04	9.61E-06	7.70E-04	1.17E-04	4.74E-05	4.34E-07	1.35E-05
**Subject 7**	3.31E-02	1.67E-07	1.75E-07	1.00E-04	3.65E-04	4.58E-05	3.60E-04	9.47E-04
**Subject 8**	2.83E-05	3.61E-08	9.36E-06	1.94E-10	2.12E-10	4.81E-05	8.30E-07	9.02E-08

### Cross-correlation between STA and raw EMG

**Figure 5:**
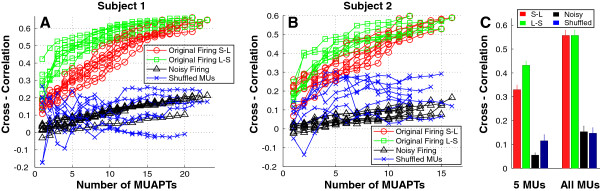
**Cross-correlation between MUAP trains and raw EMG. A**: Cross-correlations between EMG and sequentially added MUAP trains using original firings from small to large MU indices are shown in circles. Cross-correlations calculated using original firings from large to small MU indices are shown in squares. Cross-correlations calculated using noisy firings (*SD* of noise = 0.1 * *SD* of ISIs) are shown in triangles. Cross-correlations calculated using shuffled STA templates are shown in crosses. **B**: Cross-correlation results of a second subject. The symbol notations are the same as in subject 1. **C**: Cross-correlations when the first 5 MUAPTs were added together and when all MUAPTs were added. Error bars represent the standard errors across all the subjects.

The cross-correlation function values between the raw EMG signal and MUAP trains constructed using STA MUAP estimates are shown in Figure 5. Each line trace represents one individual trial. For the first subject (Figure 5A), when the MUAP trains were combined from early to late recruited MUs (MU index from small to large), the cross-correlations (open *circles*) increased almost linearly from 0.1 up to 0.66. When the MUAP trains were combined from late to early recruited MUs (MU index from large to small), the cross-correlations (open *squares*) also increased monotonically from 0.2 up to 0.66. Because the later recruited MUs tends to be larger units, as expected from the size principle, [21], and these units contains more information on the raw EMG compared with the earlier recruited smaller ones, the cross-correlation functions (open *squares*) had initially high values and rose at a faster rate than the open *circles*, but eventually plateaued at a value of 0.66. The different trials from 20% to 50% MVC showed similar cross-correlation function results.

In contrast, when the MUAP train was constructed using noisy firing times (*SD* of noise = 0.1 * *SD* of ISIs), the cross-correlations (*triangles*) were below 0.2 even after all the MUAP trains were combined. Similarly, when the MUAP templates were randomly shuffled between the various identified MUs during the construction of MUAP trains, the cross-correlations (*crosses*) fluctuated between −0.2 to 0.2.

The second subject (Figure 5B) demonstrates similar results. The cross-correlations (open *circles* and *squares*) increased from 0.06 up to 0.6 when the MUAP trains were constructed using the original firing times and STA estimated templates. When the firing times (*triangles*) or the action potential templates (*crosses*) were disturbed, the cross-correlations were below 0.3. As for subject 2, the initial increase of cross-correlation in the shuffled template condition is likely due to the fact that some MUAPs shared similar shapes in the particular electrode configuration (position and orientation over the FDI muscle). As a result, the shuffling of MU templates has only a moderate effect on the changes in correlation.

To quantify these observations across these four conditions of all the 8 subjects, the cross-correlation at each condition was summarized when the first 5 MUAP trains had been added and was compared with the cross-correlation when all the MUAP trains had been added (Figure 5C). A two-way ANOVA [(4 MUAP train formation conditions (small-large, large-small, noisy firing, and shuffled MUs) × 2 MUAP train numbers (5 MUs and all MUs)] was performed on the correlation values. A significant interaction between the MUAP train formation condition and the MUAP train number was evident (*p* < 0.05). Post-hoc pairwise multiple comparisons with Bonferroni’s correction method were then performed to examine the changes between individual conditions. Specifically, the correlation value was significantly higher when MUAP trains were added based on original firings than based on noisy firings and shuffled MUs (*p* < 0.05). When 5 MUAPs were added, the correlation value was significantly higher when MUAP trains were added from large to small than from small to large (*p* < 0.05).

## Discussion

The objective of this study was to assess the overall reliability of a sEMG discrimination method based on MU template recognition, and the general validity of placement of the accompanying spike trains that emerge as the output of the high-yield sEMG decomposition algorithm [10].

We performed two different tests to assess the validity of the decomposition of the sEMG recorded from the FDI muscle. In the first series of tests, the spike-triggered averaging (STA) technique was used to extract the action potential features from the raw recorded EMG utilizing the individual MU event times output from the decomposition algorithm. Subsequently the MU timings were altered and the STA analysis was repeated. The results show that the MUAP shape features are lost i.e., the correlation between the STA derived template and the decomposition template declines and the coefficient of variation of the STA estimated template increases, even for a small amount of added timing error. To our surprise, the change in the STA features was not sensitive to the amount of added timing noise. Meanwhile, there are also MUs that show minimal change in STA features when different amount of noise is added, possibly due to inaccurate firing identification or excessive superposition.

In the second set of tests, a cross-correlation analysis was performed between the recorded EMG signal and MUAP trains constructed using the STA derived MUAP estimates combined with the algorithm derived MU timings. The results reveal that the cross-correlation index between the raw EMG and constructed MUAP trains increases monotonically when more MUs are added to the MUAP trains; however, this increment is absent when the firing times or the MUAP templates were altered, which suggests that the firing times and estimated MUAPs are valid.

### Changes of STA due to timing noise

We did not expect that a small shift of the spike timing (by 0.6-2 ms) could substantially change the properties of the STA derived MUAP estimates. Such a high sensitivity of the STA estimates to the timing errors can arise from the particular characteristics of the recorded action potentials. Specifically, the diameter of the recording electrodes is 0.5 mm and the inter-electrode distance is 3.6 mm (center to corner electrodes) or 5 mm (corner to corner electrodes). This electrode design gives rise to sharp action potential waveforms that are broadly comparable to some recorded intramuscular EMG signals; namely the peak-peak duration of the action potential in FDI muscle is approximately 2–3 ms (Figure 2B), and the main lobe of the action potential is approximately 5 ms. Therefore, the action potential characteristics can give rise to the high sensitivity of STA to spike timing positioning.

In the STA process, when different sharp action potentials from the same MU are aligned based on the spike timing, a 0.6-2 ms of timing shift can lead to alignments of different phases of the action potentials, and ultimately lead to amplitude cancellation as shown in Figure 2B. The same reasoning can also explain the fact that the changes in the STA features are not sensitive to the amplitude of timing noise. If a 0.6-2 ms of timing shift can lead to complete amplitude cancellation, a larger degree of timing shift is unlikely to worsen the condition; as a result, similar effect of the different amount of timing noise is observed.

The significant changes of the STA features due to added timing noise suggests that the decomposed firing times are reliable for most of the MUs (Table 1). Conversely, there are MUs that show poor features extracted from our STA estimates (low correlation and high variability) computed using the original firing times (e.g., MU 4, 6, 7, and 8 in Figure 3A), and the timing noise has little effect on the STA derived features of these MUs. These results suggest that if the decomposed firing times are inaccurate the timing noise will not change the STA features, which confirms our prediction that the MUs with high correlation and low variability have reliable MUAP shapes as well as firing times. Based on these results, we can set MU selection criteria; namely, the MUs that have a correlation higher than 0.7 (the upper bound calculated from the noisy firings) and a CV smaller than 0.2 (the lower bound calculated from the noisy firings) can be retained (Table 1) for further analysis.

### Are these algorithms based on circular reasoning?

Skeptics could argue that since our STA estimates are derived from the sEMG signal using the decomposed MU firing times, that emergence of a consistent shape and a high correlation with a decomposition based template is inevitable. Therefore, the assembled STA features do not validate the accuracy of the identified firing times.

We believe that it is inevitable only if the decomposition algorithm produces a valid output, or a valid set of MUAP trains. First, the STA estimated action potential shape disappears when intentionally erroneous spike timings are used as the triggering signals. When a small (almost invisible from Figure 2A) amount of timing noise is injected to the firing times, the STA estimates do not represent the action potential shape features as shown in Figure 2B. These results suggest that the STA features are highly sensitive to spike timing errors. Second, STA does not always give rise to consistent action potential shapes. For example, the STA features are poor (i.e., low correlation and high variability) for some MUs, possibly due to spike identification errors (although extensive superposition can also be a factor), affirming that the STA shape estimate is not automatic. By setting up selection criteria, the STA analysis can exclude the identified MUs with possible errors in spike timings.

Finally, as explained in the Introduction, the decomposition algorithm derives MUAP template shapes from the uncontaminated instances of individual MUAPs and updates the shapes as they vary across the trial, and a weighted average of these variations is output as the MUAP template. The event times are based on the identification of the occurrences of the original shape and its variations. We are then working backwards, to use the event times and derive the shapes. As we have shown in this study, if there are significant errors in the identification of MUAP event times, then we will not be able to derive the STA MUAP shape that is highly correlated with the decomposition based template.

### Cross-correlation between raw EMG and MUAP trains

The combined MUAP trains from the decomposed MUs should represent the raw EMG signals, provided that the firing times and AP estimates are accurate. Indeed, our results show that the cross-correlation between the EMG signal and the reconstructed MUAP trains increases monotonically with an increasing number of MUAP trains. When the MUs were added based on the recruitment order (small to large), the cross-correlation was initially small and increased in a smaller rate than the condition where the MUs were added in a reverse order (large to small), likely because large MUs contain more correlated information with the raw EMG signal. These results suggest that the decomposed firing times and MUAP templates are reliable. Our results are further confirmed by shifting the firing times by a small amount (approximately 0.6-2 ms) and by randomly shuffling the MUAP templates between MUs during the construction of MUAP trains. The cross-correlations in these perturbed condition are considerably lower, suggesting that the perturbed MUAP trains do not represent the raw EMG signal any more.

The cross-correlation calculated based on the original firings and templates tends to plateau around 0.6 – 0.7, which is lower than 1. This effect can arise from several factors. First, only the MUs that passed the selection criteria were used to construct the MUAP trains, and the excluded MUs may still contain information of the EMG signal. Second, during the decomposition, not all the MUs embedded in the raw EMG are identifiable by the algorithm [16], especially for the small MUs under the influence of baseline noise and extensive superposition. Therefore, the residual of the EMG also contains information of the EMG signal. Lastly, the STA estimates give a single average waveform, and the action potential waveforms in a particular MUAP train are all identical. However, the action potential shape may change slightly spike-by-spike in the real signal [22,23]. This simplification of the reconstructed signal can also limit the cross-correlation values. Nevertheless, a cross-correlation around 0.6-0.7 is still relatively high, considering that all the combined MUAP trains only account for partial energy of the EMG signal and that the interference EMG has substantial superposition in relatively moderate to high force levels (20-50% MVC).

### Limitations

The series of tests used in this study affirm the general validity of the decomposed MUs; however, the accuracy of the timing for a particular firing event of a MU train is not assessed. In MU studies, the mean firing rate or the average ISI of a time segment is typically the variable of interest [24]. The STA approach in the current study provides a general assessment of the decomposed spike trains and ensures the reliability of the estimated variables (e.g., mean firing rates).

One way to assess the accuracy of individual firing events is the two-source method [12,17,24]. In this method, sEMG and intramuscular EMG are recorded simultaneously, and the decomposition results of the sEMG and intramuscular EMG signals are compared. While this method provides distinct examples of the algorithm reliability and can be used to compute accuracy, the two-source method has limitations when compared to the current validation method. For example, the muscle force is constrained to very low levels to avoid superposition such that the decomposition results of the intramuscular EMG can be trusted. As a result, the accuracy of the sEMG decomposition at moderate to high force levels (the more challenging condition for the algorithm) is unknown. Indeed, the current study provides the accuracy assessment of the decomposition in these more challenging conditions. The two-source method validates a small subset (i.e., a small fraction) of MUs decomposed by the sEMG algorithm, and the accuracy of the uncommon MUs is unknown. Similarly, one cannot use the two-source method in a daily basis when using the sEMG decomposition system. It is well known that the signal quality (e.g., the background noise level or the signal stability recorded in clinical populations) can influence the reliability of the decomposition results. Hence, the accuracy of the decomposition in different conditions cannot be guaranteed even after the algorithm has been validated by the two-source method in one condition. In contrast, the STA analysis provides a potential tool and allows us to routinely assess the overall accuracy of all the decomposed MUs at any force levels recorded in different conditions. Nonetheless, the two-source validation is still necessary in future studies to examine the accuracy of individual spike timings in order to fully confirm the decomposition accuracy.

Our STA analysis detects spurious firings (i.e., identified firings that do not exist in the real spike train) and position errors (i.e., inaccurate placement of spike timing) in the decomposition outputs. In contrast, a spike omission will not influence the STA estimate, because that action potential is missed and will not be taken into account during the averaging calculation. Therefore, the frequency of spike omission in a MUAP train is not directly assessed here. However, the spurious firings are as frequent as spike omissions for a particular MUAP train [15,17]. In a recent simulation study, we have found that the STA estimates start to deteriorate when 6% of spurious events are added to the spike trains [19]. As a result, when a large number of spike omissions occur, the equally frequent spurious firings can lead to changes in STA features, and, subsequently, the MU can be excluded based on the selection criteria (e.g., correlation coefficient < 0.7 and CV of amplitude > 0.2).

## Conclusions

This study used two sets of analyses based on STA estimates to evaluate the overall validity of a sEMG decomposition method. Our results reveal that the identified firing times and estimated MUAPs are reliable in sEMG signals recorded from FDI muscles at low to moderate force contraction levels. Additionally, the STA analysis provides a tool to detect possible identification errors and select reliably decomposed MUs from MU decomposition algorithms in general.

## Abbreviations

EMG: Electromyogram; sEMG: Surface electromyogram; STA: Spike triggered averaging; MU: Motor unit; MUAP: Motor unit action potential; FDI: First dorsal interosseous; MVC: Maximum voluntary contraction; P-P: Peak-peak; CV: Coefficient of variation; SD: Standard deviation; ISI: Inter-spike intervals; ANOVA: Analysis of variance.

## Competing interests

The authors declared that they have no competing interests.

## Authors’ contributions

All the authors have made substantial contributions to conception and design, acquisition, analysis and interpretation of data; have been involved in drafting and revising the manuscript; and have given final approval of the version to be published.
